# Genetic variation, phylogenetic relationship and spatial distribution of *‘Candidatus* Phytoplasma ulmi’ strains in Germany

**DOI:** 10.1038/s41598-020-78745-w

**Published:** 2020-12-14

**Authors:** B. Schneider, B. Hüttel, C. Zübert, M. Kube

**Affiliations:** 1 Thuenen-Institute of Forest Genetics, 15377 Waldsieversdorf, Germany; 2 Max Planck Institute for Plant Breeding Research, Max Planck Genome Centre Cologne, 50829 Cologne, Germany; 3 Department of Integrative Infection Biology Crops-Livestock, University of Hohenheim, 70599 Stuttgart, Germany

**Keywords:** Molecular biology, Bacteriology, Pathogens

## Abstract

A recent survey in Germany revealed the wide presence of ‘*Candidatus* Phytoplasma ulmi’ in native elm stands. Accessions were studied for their genetic variability and phylogenetic relationship based on the conserved *groEL* and the variable *imp* gene. While the *groEL* sequences revealed a high intraspecific homology of more than 99%, the homology of the *imp* gene dropped to 71% between distantly related sequences. Twenty-nine *groEL* and 74 *imp* genotypes were distinguished based on polymorphic sites. Phylogenetic analysis of the *groEL* gene clustered all ‘*Ca*. P. ulmi’ strains and separated them from related phytoplasmas of the 16SrV group. The inferred phylogeny of the *imp* gene resulted in a different tree topology and separated the ‘*Ca.* P. ulmi’ genotypes into two clusters, one closely related to the *flavescence dorée* phytoplasma strain FD-D (16SrV-D), the other affiliated with the *flavescence dorée* phytoplasma strains FD-C and FD70 and the alder yellows phytoplasma (16SrV-C). In both phylograms, ‘*Ca.* P. ulmi’ genotypes from Scots elm trees formed a coherent cluster, while genotypes from European white elms and field elms grouped less strictly. The regional distribution pattern was congruent for some of the *groEL* and *imp* genotypes, but a strict linkage for all genotypes was not apparent.

## Introduction

Phytoplasmas are obligate vector-borne bacterial parasites associated with diseases of more than 1000 plant species^1,2^. They form the monophyletic taxon '*Candidatus* Phytoplasma' and are members of the class Mollicutes^3^. ‘*Candidatus* Phytoplasma ulmi’ belongs to a diverse cluster of related phytoplasmas within this taxon commonly referred to as ‘elm yellows’, or the ‘16SrV group’. Members of this group are homogeneous in respect to their 16S rRNA sequence but can be divided into subgroups A to E based on restriction fragment length polymorphism analyses. In regard to affected plant species, insect vectors and ecological niches, the group is heterogeneous^4^. Beside the eponymous elm yellows, plant diseases such as *flavescence dorée*, rubus stunt, alder yellows and jujube witches’ broom are associated with phytoplasmas in this group^5–8^.

‘*Ca.* P. ulmi’ is associated with elm yellows, a serious disease of elms with a high mortal impact on populations, particularly in the eastern United States, where it was first reported in the last century^9^. Since then, elm yellows has been reported from a number of countries. Italy was the first country in Europe, but others followed^10–14^. In Germany, the disease was first reported from a Scots elm tree displaying witches’ broom symptoms^11^. This singular finding, however, gave no information on the general occurrence of the disease and the distribution of the pathogen in the German territory. It took more than 20 years until a local survey demonstrated the presence of ‘*Ca.* P. ulmi’ in European white elm trees in East Germany^13^, and the fact that all phytoplasma-positive trees lacked typical disease symptoms prompted a nationwide survey in native elm habitats and revealed an average infection rate of 28%^15^. This study also corroborated the observation of the previous survey that symptoms are rare, a circumstance explaining why the infections went unnoticed for such a long time. The situation in southern Europe is different. There, ‘*Ca.* P. ulmi’ infections seem to be associated generally with typical disease symptoms like witches’ brooms, leaf yellowing and premature leaf fall^16,17^, albeit the typical destructive phloem necrosis that American elm species display is not present in European elm species^18,19^. This decisive difference and the lack of molecular evidence were the determining factors in classifying this phytoplasma as a quarantine pathogen for the EPPO region^20^. Information on the distribution of elm yellows in Europe, which accumulated in the meantime, and the accruing molecular evidence of genetic relatedness between American and European ‘*Ca.* P. ulmi’ strains, prompted the EU to offset this classification for continental Europe^21^.

The first molecular evidence of a close relationship between American and European strains came from Southern blot hybridisation experiments using random DNA probes^7^, supported by restriction fragment length polymorphism (RFLP) and sequence analyses of the 16S rRNA gene thereafter^4^. Sequence homology of the 16S rRNA gene, together with particular features of the plant host and insect vector, led to the proposition of the novel taxon ‘*Candidatus* Phytoplasma ulmi’^3,4^. However, a closer look at genetic homogeneity with less conserved genes such as *map*, *uvrB*-*degV*, *secY* and *rpl22*-*rps3* revealed a greater intraspecific heterogeneity than expected from 16S rRNA data. A Serbian study of ‘*Ca.* P. ulmi’ accessions distinguished five genotypes with significant differences in relation to the American-type strain EY1^T^ based on *map*, *secY* and *rpl22*-*rps3* genes^22^. A combination of polymorphic sites of the same genes even distinguished 30 genotypes in a Croatian study^17^. Beside the taxonomically fundamental 16S rRNA gene, the *groEL* gene was proposed as an additional marker to resolve taxonomic issues in complex bacterial groups which has the ability to ensure accurate predictions and to replace more time intense multiple gene analyses. It has been demonstrated that a 550 bp *groEL* fragment can predict relationships within *Thermoanaerobacter* species with the same accuracy compared to a combinatorial analysis of three genes^23^. Sequence data for this gene were successfully used to differentiate subgroups further within the aster yellows, or 16SrI group, and the Bermuda grass white leaf, or 16SrXIV group^24,25^. A recently installed Web interface enables the comparison of *groEL* sequences from different taxonomic phytoplasma entities^26^, although the numbers of data are still low. In contrast to conserved genes, sequence-variable genes like the immunodominant membrane protein (*imp*) gene have only been used to a limited extent in the 16SrII (‘*Ca*. P. aurantifolia’)^27^ or 16SrX (apple proliferation)^28,29^ groups to resolve intraspecific variation, phylogenetic linkages and geographical distribution.

In this study, we examine genetic variations, the phylogenetic relationship and the regional distribution of ‘*Ca.* P. ulmi’ accessions with a *groEL* gene fragment and the *imp* gene—two markers of different resolving powers adding specific information to survey results published recently by the authors.

## Results

### DNA amplification of *groEL*- and *imp* fragments

**Figure 1:**
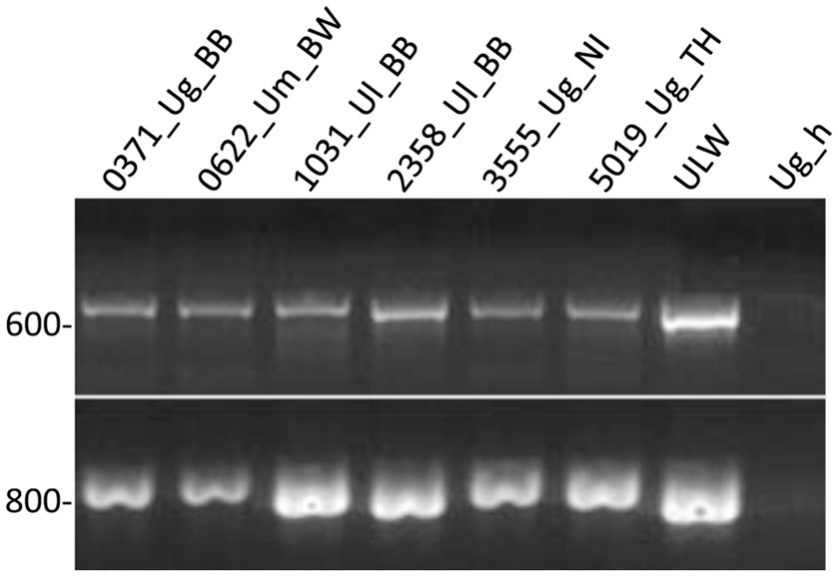
Amplification of *imp* (upper panel) and *groEL* (lower panel) fragments from ‘*Ca.* P. ulmi’ accessions. Only examples are shown. Sample names are indicated on the top, including the host plant and Federal State. Ug_h, DNA from a healthy Scots elm accession. The gel was photographed with a VWR Imager 2 System, Software Version 1.5.6.0 (https://de.vwr.com) and cropped with Photoshop CS3 (www.adobe.com).

The selected forward and reverse primers for the *groEL*- and *imp* genes amplified fragments of about 880 bp and 675 bp, respectively, from all 288 ‘*Ca.* P. ulmi’ accessions (Fig. 1). The primers also amplified *groEL*- and *imp* fragments from the phytoplasmas alder yellows strain ALY, the ‘*Ca.* P. ulmi’ strain ULW and the *flavescence dorée* strain FD70.

### Genetic variation in the ‘*Ca.* P. ulmi’ *groEL* gene sequences

Homology among the ‘*Ca.* P. ulmi’ *groEL* sequences, including the ‘*Ca.* P. ulmi’ strain ULW, ranged between 99 and 100%, while homology to ALY (MT638097), FD70 (MT638098) and ‘*Candidatus* Phytoplasma ziziphi’ sequences ranged between 94% and 96.4%. Accession 4120_Ul_SN (MT638093), isolated from a European white elm tree, shared a lower sequence homology (95.7% to 96.4%) to all other ‘*Ca.* P. ulmi’ sequences and was closer related to the ALY (99.1%) and FD70 (98.6%) sequences. This accession was included in the phylogenetic analysis but not considered as a sensu stricto member of the species ‘*Ca.* P. ulmi’. Homology to the distantly related ‘*Ca*. Phytoplasma mali’ *groEL* sequence was on average 69.7%.

The multiple alignment of the 790 bp *groEL* fragment from the ‘*Ca.* P. ulmi’ sequences, excluding sequence 4120_Ul_SN, revealed a total of 18 polymorphic sites, of which 12 caused a nonsynonymous substitution (Table 1). However, mutations altering the amino acid in a significant number of isolates were only present at positions 18, 249 and 438. The remaining non-silent mutations occurred in genotype groups with a low number of isolates.

**Table 1:** Nucleotide position and variation thereof in the 790 bp *groEL* fragment of ‘*Ca.* P. ulmi’ accessions.

Position^a^	Reference nucleotide and amino acid^b^		A^c^		C		G		T	
**18**	A	(N)	–		1	(H)	–		11	(Y)
26	T		–		81		–		–	
47	A		–		–		–		81	
71	C		105		–		39		–	
109	G	(S)	1	(N)	–		–		–	
224	A		–		–		88		–	
**249**	G	(A)	–		–		–		94	(S)
262	C	(T)	2	(K)	–		–		–	
290	C		–		–		–		67	
342	A	(Y)	–		–		2	(C)	–	
399	G	(D)	2	(N)	–		–		–	
**438**	T	(S)	114	(T)	–		–		–	
458	C	(F)	–		–		4	(L)	–	
538	C	(A)	–		–		2	(V)	–	
733	T	(M)	–		4	(T)	–		–	
767	C		1		–		–		108	
770	A	(Q)	–		–		–		2	(H)
771	A	(I)	–		–		–		2	(F)

^a^Position of mutation relative to the first nucleotide for database entry MT638069. The underlined numbers indicate non-silent mutations. Bold numbers indicate mutations at positions causing an amino acid (aa) exchange in a significant number of samples.

^b^Prevalent nucleotide and encoded aa (in bracket) when different to other genotypes.

^c^Number of sequences with a different nucleotide at the indicated position and altered aa (in brackets), where applicable. The total number of sequences considered was 287.

A comparison of the 288 *groEL* sequences, including accession 4120_Ul_SN, on the basis of complete sequence homology, differentiated 29 genotypes, of which 18 occurred more than once (Table 2). The genotype groups correlated in most cases with the elm species from which they were isolated. In cases where the ‘*Ca.* P. ulmi’ genotype was associated with a different elm species, more than one elm species grew at the sampling location. However, if fruits were unavailable or leaf shapes difficult to interpret, a misidentification of the elm species cannot be excluded.

**Table 2:** ‘*Ca.* P. ulmi’ genotypes distinguished based on complete *groEL* fragment homology.

Genotype^a^/sum	Prevailing elm species	No. of sequences	Acc. No.^b^	Other elm species^c^		
				*U. glabra*	*U. laevis*	*U. minor*
I	*U. glabra*	67	MT638090	–	1	2
II	*U. minor*	61^d^	MT418907	2	6	–
III	*U. glabra*	36	MT638073	–	1	2
IV	*U. laevis*	35	MT638072	1	–	5
V	*U. laevis*	34	MT638096	3	–	2
VI	*U. glabra*	6	MT638095	–	–	–
VII	*U. glabra*	6	MT638071	–	–	1
VIII	*U. glabra*	5	MT630879	–	–	–
IX	*U. laevis*	4	MT638088	–	–	1
X	*U. minor*	4	MT638091	2	–	–
XI	*U. laevis*	4	MT638092	–	–	–
XII	*U. glabra*	3	MT638078	–	–	–
XIII	*U. glabra*	3	MT638069	–	–	–
XIV	*U. glabra*	2	MT638094	–	–	–
XV	*U. glabra*	2	MT638074	–	–	–
XVI	*U. minor*	2	MT638083	–	–	–
XVII	*U. glabra*	2	MT638087	–	–	–
XVIII	*U. glabra*	2	MT638076	–	–	–
Sum		278		8	8	13

^a^Only genotypes which occurred at least twice are listed.

^b^Accession number of a representative group member.

^c^Number of isolates not associated with the prevailing elm species.

^d^The number includes the ‘*Ca.* P. ulmi’ strain ULW.

### Genetic variation in the ‘*Ca.* P. ulmi’ *imp* gene sequences

The length of the ‘*Ca.* P. ulmi’ *imp* gene varied. While the majority of isolates (N = 253) showed a gene length of 465 bases, 34 isolates had a gene six bases larger. The *imp* gene of accession 4120_Ul_SN (MT668488) was shorter by six bases, a feature shared with the phytoplasma strain ALY. Homology among the ‘*Ca.* P. ulmi’ *imp* gene sequences ranged from 71 to 100%, whilst homology to the *imp* gene of the 16SrV-D group phytoplasma FD-D ranged between 66 and 79%, and homology to the 16SrV-C group sequences of ALY (MT668499), FD70 (MT6684500) and FD-C ranged between 67 and 71%. Homology to the 16SrV-B subgroup member ‘*Ca*. P. ziziphi’ was generally lower with a narrower bandwidth and ranged between 61 and 65%. The accession 4120_Ul_SN shared, as evidenced with the *groEL* gene, higher homology with 16SrV-C phytoplasmas (ALY, 83% and FD70, 92%) than with the ‘*Ca.* P. ulmi’ accessions (≈ 71%). Homology of the ‘*Ca.* P. ulmi’ strains to the distantly related species ‘*Ca*. P. mali’ was about 42%.

**Figure 2:**
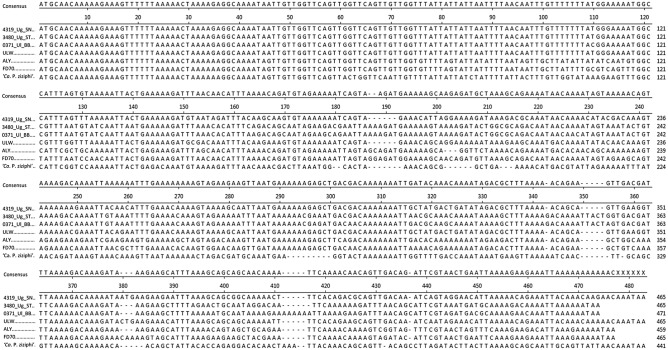
Alignment of *imp* sequences from 4319_Ug_SN (MT668492), 3486_Ug_ST (MT668480), 0371_Ul_BB (MT668429), ULW (MT418908) and the 16SrV-group phytoplasmas ALY (MT668499), FD70 (MT668500) and ‘*Ca.* P. ziziphi’ (CP025121) with Clustal W. The consensus sequence is given above the alignment, showing bases represented in more than 50% of sequences. Sequence alignments have been performed with Clustal W (www.ebi.ac.uk/clustalw/).

The multiple alignment of 287 ‘*Ca.* P. ulmi’ sequences revealed a complete sequence identity within the first 60 nucleotides, and only three sequences showed one mismatch each over the next 30 bases (Fig. 2 and data not shown). The conserved 5′ stretch was reduced to 34 bases when the *imp* genes of accession 4120_Ul_SN, and from the phytoplasmas ALY, FD70; FD-C, FD-D and ‘*Ca*. P. ziziphi’, were included (Fig. 2 and data not shown). After position 120, the ‘*Ca.* P. ulmi’ *imp* sequences became highly variable and formed repeated gaps in the alignment. These gaps were caused by aligning sequences, which belonged to three different categories. The largest group comprised 237 sequences with a gene length of 465 bp and was represented by sequence MT668492 (4319_Ug_SN). The second group comprised 34 sequences with a gene length of 471 bp and was represented by sequence MT668429 (0371_Ul_BB). The last group comprised 16 sequences with a gene length of 465 bp, represented by sequence MT668480 (3480_Ug_ST). The alignment of ‘*Ca.* P. ulmi’ sequences of one category created alignments without gaps. The *imp* sequence of sample 4120_Ul_SN did not align to any sequence without gaps.

Based on complete sequence identity, 74 genotypes were distinguished among the 288 ‘*Ca.* P. ulmi’ accessions. Twenty-eight genotypes were represented by more than one sequence (Table 3), and 46 genotypes were unique and their electropherograms re-assessed when a single nucleotide mismatch defined their group exclusion. The *imp* genotypes grouped, with few exceptions, like the *groEL* genotypes according to the elm species from which they originated. The *imp* genotype I comprised most of the accessions from Scots elm trees, and the gene showed higher conservation compared to the linked *groEL* genes, which associated the remaining 44 accessions to 14 *groEL* genotype groups.

**Table 3:** ‘*Ca.* P. ulmi’ groups established on complete homology of the *imp* gene.

Genotype^a^/sum	Prevailing elm species	No. of sequences	Acc. No.^b^	Other elm species^c^		
				*U. glabra*	*U. laevis*	*U. minor*
I	*U. glabra*	101	MT668492	–	9	3
II	*U. minor*	26^d^	MT418908	1	3	–
III	*U. glabra*	15	MT668459	–	–	–
IV	*U. laevis*	14	MT668429	2	–	1
V	*U. laevis*	9	MT668462	–	–	3
VI	*U. laevis*	9	MT668464	1	–	–
VII	*U. laevis*	9	MT668445	–	–	1
VIII	*U. minor*	9	MT668456	–	1	–
IX	*U. minor*	8	MT668475	–	–	–
X	*U. glabra*	4	MT668444	–	–	–
XI	*U. glabra*	4	MT668434	–	–	1
XII	*U. minor*	3	MT668437	–	–	–
XIII	*U. laevis*	2	MT668453	–	–	1
XIV	*U. laevis*	2	MT668483	–	–	–
XV	*U. glabra*	2	MT668435	–	–	–
XVI	*U. glabra*	2	MT668497	–	–	–
XVII	*U. glabra*	2	MT668482	–	–	–
XVIII	*U. glabra*	2	MT668494	–	–	–
XIX	*U. glabra*	2	MT668458	–	–	–
XX	*U. minor*	2	MT668446	–	–	–
XXI	*U. laevis*	2	MT668498	–	–	–
XXII	*U. glabra*	2	MT668447	–	–	–
XXIII	*U minor*	2	MT668485	–	–	–
XXIV	*U. laevis*	2	MT668440	–	–	–
XXV	*U. minor*	2	MT668442	–	–	–
XXVI	*U. minor*	2	MT668433	–	–	1
XXVII	*U. glabra*	2	MT668448	–	–	–
XXVIII	*U. glabra*	2	MT668480	–	–	–
Sum		242		4	13	11

^a^Only genotypes which occurred at least twice are listed.

^b^Accession number of a representative group member.

^c^Number of isolates not associated with the prevailing elm species.

^d^The number includes ‘*Ca.* P. ulmi’ strain ULW.

The majority of ‘*Ca.* P. ulmi’ accessions (N = 254), the strain ULW and FD70 coded for a protein of 154 amino acids (aa). Thirty-four accessions, mostly from European white elms (N = 26), including the strain FD-D, encoded a protein two aa larger. The *imp* gene of accession 4120_Ul_SN coded for a protein of 152 aa only, a feature shared with ALY. Due to the high sequence heterogeneity between the ‘*Ca.* P. ulmi’ protein sequences, homology between distantly related isolates dropped to 48%. A protein-based reassessment of the genotype groups revealed that three sequences, which showed a single base mutation, shared identical IMP sequences to others reducing the number of genotypes to 71. Homology to the 16SrV-C group phytoplasmas ALY and FD70 ranged from 42 to 52%, to the 16SrV-D group phytoplasma FD-D from 45 to 61% and decreased below 39% in comparison to the 16SrV-B group phytoplasma ‘*Ca*. P. ziziphi’. The accession 4120_Ul_SN shared homology of 34%, 54% and 86% to the 16SrV-B, -D and C phytoplasmas, respectively.

### ‘*Ca.* P. ulmi’ phylogeny based on *groEL* sequences

**Figure 3:**
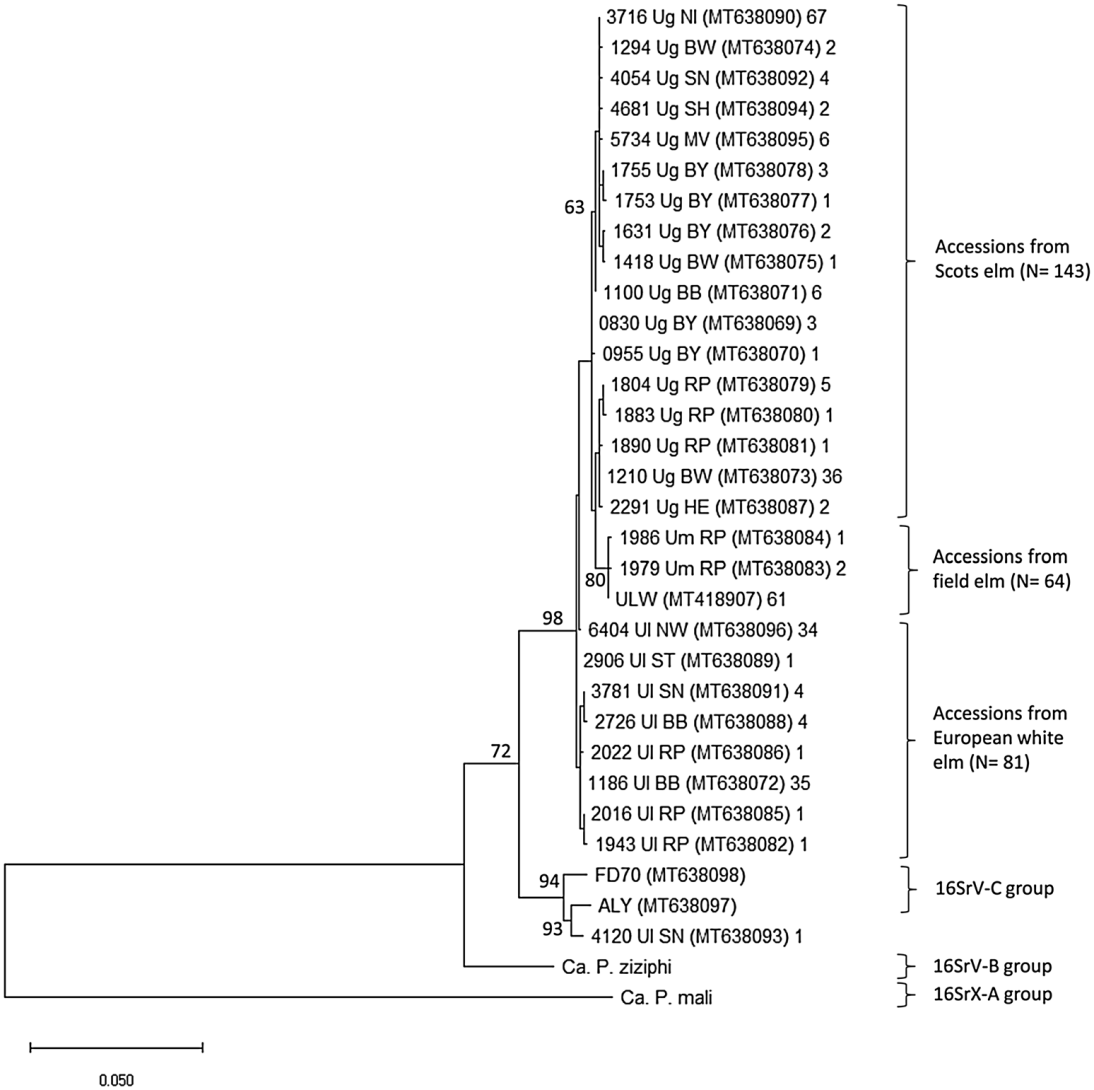
Phylogenetic tree inferred from *groEL* gene fragments, using the maximum likelihood method. All sequences, except those from ‘*Ca*. P. ulmi’ strain ULW, ‘*Ca*. P. ziziphi’ and ‘*Ca*. P. mali’, were obtained in this study. Isolate numbers are followed by a suffix indicating the original plant host species and the Federal State they were collected from. The accession numbers are given in brackets. Only one representative of each genotype was included in the analysis, but the numbers of genotype members are indicated after the brackets. A total of 790 bases were included in the dataset. Bootstrap values (> 50) for 1.000 replicates are indicated. The phylogenetic tree was compiled with the software MEGA version X (www.megasoftware.net).

The phylogeny inferred on the *groEL* gene indicated a monophyletic origin of the ‘*Ca.* P. ulmi’ accessions, except for genotype 4120_Ul_SN, which clustered with ALY and FD70 (Fig. 3). The ‘*Ca.* P. ulmi’ clade split into two sub-branches whereby the genotypes from Scots elms and European white elms formed a separate entity, and the genotypes from field elm branched off from a Scots elm sub-clade. In a preliminary analysis, two *groEL* sequences (KJ939991, KJ939992) assigned to the species ‘*Ca.* P. ulmi’ clustered with the ALY- and FD70 *groEL* sequences but not with the ‘*Ca.* P. ulmi’ sequences obtained in this work. The two sequences were not included in the final analysis, as the fragment size of ca. 550 nt reduced the number of discriminating positions and affected the tree topology (data not shown).

### ‘*Ca.* P. ulmi’ phylogeny based on *imp* sequences

**Figure 4:**
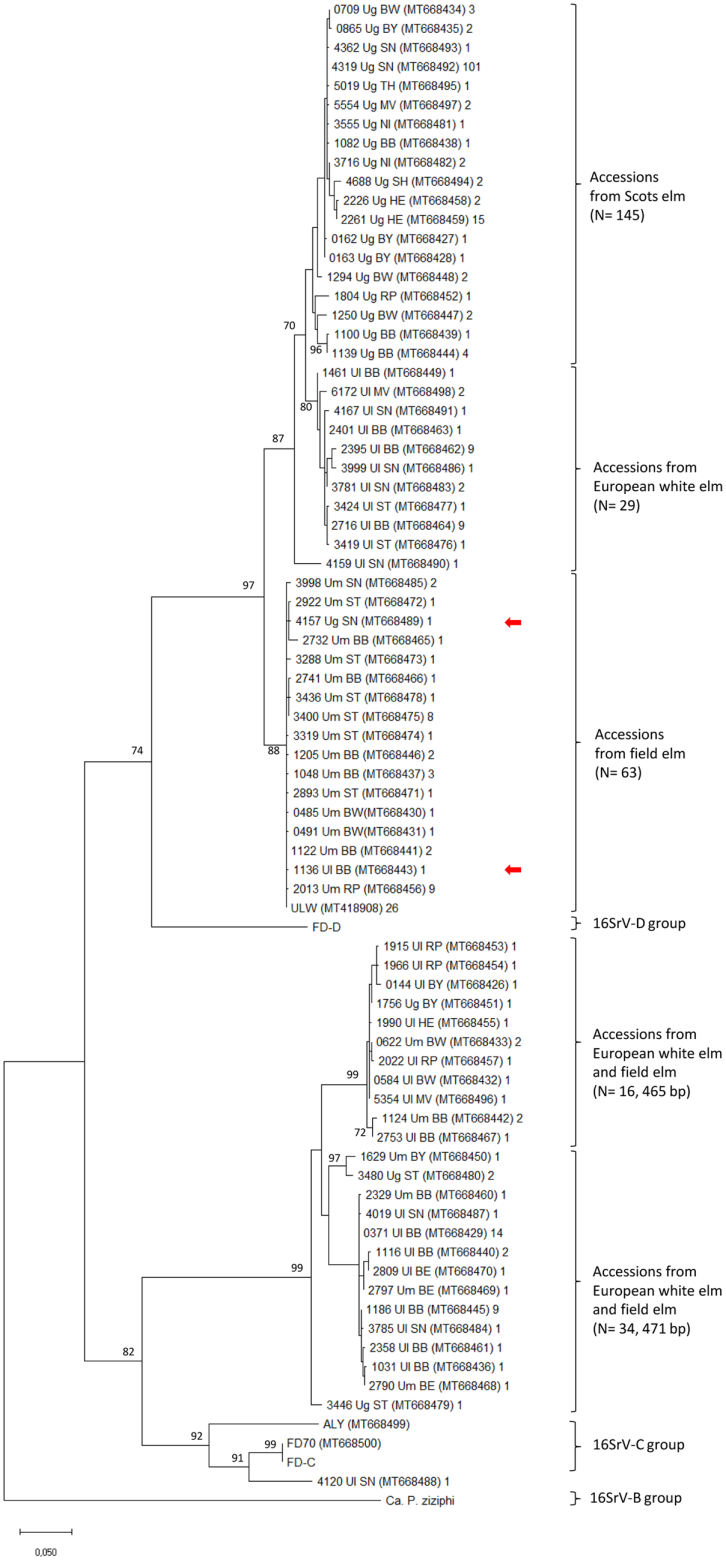
Evolutionary analysis by maximum likelihood method of the *imp* gene from 74 ‘*Ca.* P. ulmi’ genotypes and the 16SrV group phytoplasmas *flavescence dorée* strains FD-C, FD-D and FD70, ALY and ‘*Ca*. P. ziziphi’. All sequences, except those from ‘*Ca*. P. ulmi’ strain ULW, FD-C, FD-D and ‘*Ca*. P. ziziphi’, were obtained in this study. Isolate designation and additional information as given in Fig. 3. There were a total of 429 positions in the final dataset, and bootstrap values (> 50) for 1.000 replicates are indicated. Red arrows indicate accessions not originating from field elms. The phylogenetic tree was compiled with the software MEGA version X (www.megasoftware.net).

The phylogeny inferred on the *imp* gene sequences divided the ‘*Ca.* P. ulmi’ genotypes into two clades. Genotypes of each clade were affiliated with phytoplasmas of a different 16SrV subgroup (Fig. 4). The majority (N = 238) of ‘*Ca.* P. ulmi’ sequences clustered with the *flavescence dorée* strain FD-D, a member of the 16SrV-D subgroup. Genotypes of this clade split further into three branches comprising, with few exceptions (Fig. 4, red arrows), isolates of one elm species (Fig. 4, upper part). Fifty ‘*Ca.* P. ulmi’ genotypes were more closely related to phytoplasmas of the 16SrV-C subgroup, represented by the *flavescence dorée* strains FD70 and FD-C and the alder yellows phytoplasma ALY (Fig. 4, bottom part). These ‘*Ca.* P. ulmi’ genotypes separated further according to their gene length and were less strictly arranged according to the elm species of origin. The phylogenetic tree inferred from deduced protein sequences resembled the DNA-derived tree, except that the 16SrV-C subgroup phytoplasmas neighboured the 16SrV-D group strain (data not shown).

### Non-synonymous vs. synonymous mutations

The ratio between non-synonymous over synonymous mutations was 1.53 (dN = 0.23; dS = 0.15) for *imp* and 1.00 (dN = 0.01; dS = 0.01) for the *groEL*-fragment. The ratio of dN/dS > 1 for the *imp* gene indicates a positive selection, which was not the case for the *groEL*-fragment.

### Spatial distribution pattern of *groEL*- and *imp* genotypes and gene linkage

**Figure 5:**
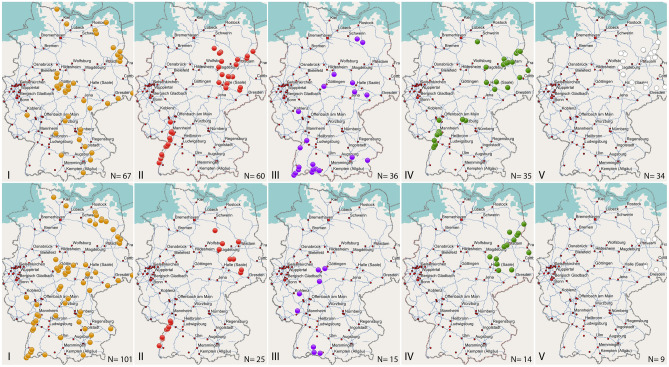
Spatial distribution patterns of *groEL* genotypes I to V (upper panel) and *imp* genotypes I to V (bottom panel). The number of sequences per genotype is indicated and illustrated as coloured dots. Due to the map scale, not all dots are visible. Maps were created with the software Garmin BaseCamp version 4.7.1 (www.garmin.com). Individual maps were assembled and labelled with the software program Photoshop CS3 (www.adobe.com).

The spatial distribution of *groEL* and *imp* genotypes was not homogeneous in the territory, and flexibility in the combination of genotype linkages was apparent. The polymorphism of both genes resulted in genotype groups with a low number of datasets, which were insufficient for conclusive results. Therefore, only the first five genotype groups comprising nine sequences or more were analysed (see Tables 2 and 3). The spatial distribution of the *groEL*- and *imp* genotypes I to V is displayed in Fig. 5, and while the *groEL* genotypes I and III were present in South, Central and East Germany, genotypes II and IV were absent in the central and the south-eastern regions (Fig. 5, top panel). *GroEL* genotype V sequences were only found in East Germany.

The distribution of the *imp* genotypes I, II and V largely matched the respective *groEL* genotype spread (Fig. 5, bottom panel), although small differences existed. The *imp* genotype I for example, was in contrast to the *groEL* genotype I, also present along the upper Rhine valley, in the far south-west of Germany. A greater disparity of distribution was observed between the ‘*Ca.* P. ulmi’ *imp* and *groEL* genotype groups III and IV. The *imp* genotype III was absent in East Germany, while the *imp* genotype IV did not occur in the south of the country.

The regional distribution of genotypes was largely congruent with the distribution of the elm species. Genotype groups I and III were mostly associated with Scots elms, genotype II with field elms and genotypes IV and V with European white elms. However, there was some flexibility in the linkage of *groEL*- and *imp* genotypes. The *imp* genotype I, comprising 101 sequences, was linked to 15 different *groEL* genotype groups, of which the *groEL* genotype group I comprised the most with 57 sequences, followed by group III with 16 sequences and group VI with six sequences. The remaining 22 sequences were part of 12 other *groEL* genotype groups. The same was true when considering the *groEL* group II in comparison with *imp* genotype group II. All 26 sequences of the *imp* genotype group II were part of the corresponding *groEL* group, but the remaining 35 *groEL* sequences were associated with a further 19 *imp* genotype groups. Neither the amplification products nor the electropherograms of the *groEL* or *imp* sequences provided evidence that the amplimers consisted of polymorphic fragments. The observed gene combinations were therefore considered as a true linkage and the result of selective evolutionary pressure.

## Discussion

A recently conducted survey demonstrated the presence of ‘*Ca.* P. ulmi’ in many parts of Germany, but the genetic variation of the regional strains and their phylogenetic relationship were not examined. In this work, we address both issues through the sequence analyses of two genes. The conserved *groEL* gene was selected to provide a better intraspecific resolution, due to a higher sequence variation compared to the 16S rRNA gene^24,25,30^, and the variable *imp* gene was chosen to tap the full range of genetic polymorphism of a variable gene.

The primer sets for amplifying both genes were deduced from the database entries of the respective genes from the ‘*Ca.* P. ulmi’ strain ULW. The oligonucleotides were compared to target genes of close relatives from the 16SrV-B, -C and -D subgroups, where available. Due to the high conservation of the *groEL* gene and the likelihood of an unspecific amplification of other bacterial *groEL* genes from crude plant extracts, the reverse primer showed six mismatches to the ‘*Ca.* P. ziziphi’ sequence, the only other full-length *groEL* gene in the 16SrV group published. Primers for the amplification of the *imp*-*pyrG* region were fully homologous to the respective sequences of ‘*Ca.* P. ulmi’ strain ULW and the *flavescence dorée* strain FD-C. Both primer sets enabled the amplification of the target fragments from all ‘*Ca.* P. ulmi’ accessions, including the strains FD70 and ALY. Although, the sequencing results revealed no evidence of mixed infection of plants by more than one ‘*Ca.* P. ulmi’ strain, we do not exclude this possibility. It is conceivable that other ‘*Ca.* P. ulmi’ strains were present, but they must have been present in small numbers so alternative base calls were below the threshold line of the electropherogram. A closer examination of this matter would have exceeded the scope of this work.

Previous studies have demonstrated the genetic variability of ‘*Ca.* P. ulmi’ isolates, but genotype classification was based on a combined evaluation of three to four genes^17,22^. In this work, we examined for the first time the resolving power of the *groEL* gene for intraspecific ‘*Ca.* P. ulmi’ differentiation. Although sequence polymorphism did not exceed 1%, except for accession 4120_Ul_SN, 29 genotypes were identified. Compared to the intraspecific 16S rDNA sequence heterogeneity of < 0.4%, the sole consideration of the *groEL* gene allowed a significantly higher intraspecific resolution. It is conceivable that an even greater number of genotypes would have been identified, as the examined accessions represented only 16% of the ‘*Ca.* P. ulmi’-positive samples identified in the previous survey^15^. However, samples were carefully selected based on an even geographical spread and on a representative number of samples from all three elm species.

The *groEL* and *imp* genotype groups comprised, with a few exceptions, accessions originating from the same elm species, although parallel genotype groups with the same host species existed. Taking into consideration the many sequences of the larger genotype groups, the distinct host–pathogen strain association seems not to be arbitrary. It is unlikely that the *groEL* gene product, a chaperonin, is responsible for this specificity; however, it is likely that *groEL* is correlated to one or more such genes specifying this feature.

One of these candidate genes could be *imp*. The gene, but in particular the encoded protein, is a focus of phytoplasma research and the subject of host–pathogen interaction studies and the development of immuno-diagnostics^28,31,32^. However, despite its variability, the gene has been rarely used for intraspecific sequence polymorphism studies^27–29^. In this study, we have employed for the first time the *imp* gene of ‘*Ca.* P. ulmi’ for this purpose and determined a high strain polymorphism, which was not only expressed by its sequence length, but also by its sequence composition. A calculated overall dN/dS ratio of 1.53 indicates that the ‘*Ca.* P. ulmi’ *imp* gene is subject to a high selective pressure, a factor that was demonstrated to act on other phytoplasma membrane protein genes too^33^. The high number of *imp* genotypes identified herein point in this direction, but on the other hand, the majority of sequences belonged to a few groups, and again, those groups were, as for the *groEL* gene, associated with a specific elm species. Whether or not the ‘*Ca.* P. ulmi’ membrane protein has an important function in host–pathogen recognition, as studies with other phytoplasmas suggest^28,32,34^, remains to be demonstrated, but in a recent study the interaction of the FD-D IMP with gut membrane proteins of vector species was proven^35^. That immunodominant phytoplasma membrane proteins seem to play a generally important role in the attachment to host cell membranes has been also demonstrated for AMP, a major membrane protein of aster yellows group phytoplasmas^36^. Challenging the attachment of phytoplasmas to host cell membranes in the presence of recombinant AMP or opsonating anti-AMP antibodies reduced the transmission frequency significantly for two vector species. However, the origin of the high polymorphism remains unclear. One likely notion to explain the host-genotype association might involve monophagous vectors, which specifically transmit the prevailing regional ‘*Ca.* P. ulmi’ strain. An occasional presence of this strain in other elm species might be explained by a rare probe feeding of the vector or by root anastomoses. However, as no vector or vectors have been identified in Germany, this question remains open.

The phylogenetic analysis of the *groEL* fragment placed all, except one *‘Ca.* P. ulmi*’* accession, on one root separated from members of the 16SrV-B and C subgroup. The accessions on this root were arranged according to the elm species from which they were isolated. This highly ordered elm species-related grouping broke up when shorter sequences (< 560 bp) were included in the analysis, and although these sequences did not cluster with the ‘*Ca.* P. ulmi’ accessions, the loss of discriminating characters changed the tree hierarchy. This example demonstrates that although the *groEL* gene is a proven universal target for phylogenetic analysis, a certain sequence length might be necessary to exploit the full information content of a gene. If not so, biologically significant features might be obstructed, resulting in a less meaningful phylogenetic tree.

The *imp*-derived phylogenetic tree was more complex and separated the ‘*Ca.* P. ulmi’ accessions on two separate roots. Interestingly, the accessions on both roots behaved differently. Whereas the 16SrV-D-related strains clustered according to their elm host, the 16SrV-C-related accessions were grouped according to their sequence length, rather than to their elm host. Although, the Scots elm accession and the European white elm accession which were interspersed in the 16SrV-C-related field elm (Um) branch might have been a matter of species misidentification. The breakup of a monophyletic relationship is not unique for ‘*Ca.* P. ulmi’. A similar result was obtained after comparing the *imp* genes of hypovirulent ‘*Ca*. P. prunorum’ strains, which displayed monophyly based on the *aceF-*gene^37^. This is, however, not always the case as shown for the stolbur membrane protein gene *stamp*^38^. Here, all *stamp* sequences grouped according to established *tuf* clusters. Why the immunodominant membrane protein genes of some phytoplasma groups seem to evolve independently from other genes might be explained by their interaction with host proteins, but this does not seem to apply for the *imp* sequences of the flavescence dorée strains FD-C and FD-D, which differ but share the same plant and insect hosts.

We tried to identify a superordinate clustering principle, like provenience, for arranging genotypes at the terminals of a clade. For some, like the European white elm accessions (N = 29) in the upper part of the phylogram (Fig. 4), their origin laid in the east of Germany, but for other clades and sister clades, accessions of all states assembled. Interesting to note was the position of the three *flavescence dorée* strains, FD70, FD-C and FD-D, relative to the ‘*Ca.* P. ulmi’ accessions. FD strains have been distinguished on the basis of the *map* and *uvr-degV* genes and their geographic origin^39^. Here, we could show that the *imp* gene of FD70, a strain from south-western France, was identical to the *imp* gene of FD-C, a strain restricted to Italy. Both strains were closely related to alder yellows, to accession 4120_Ul_SN and to a subset of ‘*Ca.* P. ulmi’ accessions mainly isolated from European white elm. The *imp* gene of the FD-D strain, on the other hand, was more closely related to ‘*Ca.* P. ulmi’ strains than to FD70 and FD-C. This situation demonstrates the range of genetic diversity within the 16SrV group and the complex phylogenetic relationship between its members. However, the fact that a variable gene, like *imp*, is more related between otherwise more remote strains highlights its biological importance.

The study revealed a high number of *groEL*- and *imp* genotype groups, many of which comprised too few sequences to expect significant results. The major genotype groups, however, showed distinct distribution patterns, and in many cases these patterns corresponded, albeit a certain number of accessions, either in the *groEL*- or *imp* genotype groups, were classified in non-corresponding groups. Hence, a certain flexibility of gene linkage exists, but not to a degree of complete randomness. This is evidenced by the fact that such *groEL*- or *imp* sequence variants were always associated with other *imp*- or *groEL* sequence variants of the same elm species. It also seems that another so far unknown factor determines the host–pathogen association. The delineation of elm species-specific genotypes in regions with mixed elm populations points to an insect vector as a decisive factor; indeed, monophagous phloem feeders have been identified. For example, for field elm, *Macropsis glandacea*, (syn. *Macropsis mendax*) the only experimentally confirmed vector for ‘*Ca.* P. ulmi’ in Italy^40^, and *Iassus scutellaris* have been described^41^. Furthermore, a number of polyphagous phloem-feeders such as *Allygidius atomarius* or *Empoasca vitis* have been described, but for many *Auchenorryhncha* species found on elm trees, the host specificity is not completely clear. Therefore, the initiative to identify ‘*Ca.* P. ulmi’ vectors might provide important new information on how the spatial distribution of ‘*Ca*. P. ulmi’ strains has evolved.

## Materials and methods

### Plant samples, DNA extraction and phytoplasma strains

Shoot samples from Scots elms (*Ulmus glabra*), European white elms (*Ulmus laevis*) and field elms (*Ulmus minor*) were collected from October 2017 to May 2019 in natural elm habitats in Germany. Total nucleic acids were extracted from 125 mg of phloem tissue, according to a procedure described by Ahrens and Seemüller^42^, and 288 phytoplasma-positive samples from 133 sampling sites were selected for analysis. From 18, 94 and 21 sites, one, two and up to four accessions were examined, respectively. Identification of ‘*Ca.* P. ulmi’ was performed by a pathogen-specific quantitative real-time PCR assay described by Schneider et al.^15^. DNA from the 16SrV-C group phytoplasma alder yellows, strain ALY^16^ and the 16SrV-A group phytoplasma ‘*Ca. P. ulmi*’ strain ULW (obtained by G. Morvan, INRA, France), maintained in *Catharanthus roseus*, was extracted from leaf midribs. DNA from the 16SrV-C group phytoplasma *flavescence dorée*, strain FD70^43^ maintained in *Vicia faba*, was obtained from M. Maixner, JKI, Germany. The nucleic acid pellets were resuspended in 200 µl of ddH_2_O.

### Primer design

The primer design for amplifying the ‘*Ca.* P. ulmi’ *groEL*- and *imp* fragments was based on the full-length sequence of the *groEL* (MT418907), *dnaD* (MT418910), *imp* (MT418908) and *pyrG* (MT418909) gene from the ‘*Ca.* P. ulmi’ strain ULW. Validation of the deduced primers was performed via alignment with the *groEL* gene of ‘*Ca.* P. ziziphi’ (CP025121) and the dnaD-*imp*-*pyrG* fragments of ‘*Ca.* P. ziziphi’ (CP025121) and of the *flavescence dorée* phytoplasma strain FD-C (KJ402359). All primer sequences are listed in Table 4.

**Table 4:** Amplification primers for the *groEL*- and the *imp* gene fragments.

Primer abbreviation	Sequence (5′–3′)
fEY_*groEL*	GTTAATGATGGCGTTACAATCGC
rEY_*groEL*	GTTAAAGAAGGACTTTTATCCGC
fEY_*imp*	CATTTTAAATACTGTATATTAAATAC
r*pyrG*	GACCTTTTAAACCACATCC

### Polymerase chain reaction (PCR) amplification and sequencing

PCR fragment amplification was carried out in 20 µl reactions, using 20 pmol of forward and reverse primer, 2 µl of DNA extract, 10 µl of 2 × primaQuant PCR master mix (Steinbrenner, Wiesenbach) and 4 µl of sterile water. The amplification was performed in a qTower (Analytik Jena AG, Jena) with the following cycling conditions: three minutes at 95 °C for the initial denaturation and polymerase activation, followed by 35 cycles consisting of 10 s at 95 °C, 20 s at 59 °C (*groEL*), or 54 °C (*imp*) and 45 s at 72 °C. The final elongation step was set to 5 min at 72 °C. After amplification, 2 µl of the PCR reaction mix were used to verify product yield and size by electrophoresis in a GelRed-stained 2% agarose gel. The PCR fragments of the remaining 18 µl were purified by a PCR purification kit (Qiagen, Hilden) and quantified using a Qubit fluorometer (Thermo Fisher Scientific, Darmstadt). Sequencing was performed with the forward and reverse primers used for amplification.

### Sequence analysis, phylogenetic tree construction and calculation of non-synonymous vs. synonymous mutations

The forward and reverse sequences of the PCR fragments were aligned for sequence correspondence, using the program Seqman (DNASTAR INC., Madison). Consensus sequences were used for analysis when fully matching. In the case of ambiguous base calls, sequencing was repeated. The *groEL* fragments were trimmed to a length of 790 bp for a common 5′ start and 3′ end. The 675 bp *imp*/*pyrG* fragments, hereafter referred to as *imp* fragments or genes, were trimmed to the coding region of the *imp* gene. The *groEL*- and *imp* genes from the phytoplasma strains ALY, FD70 and ULW were also sequenced, trimmed as above and included in the analysis. To avoid multiple database entries with identical sequences, the DNA sequence of one representative of each genotype group was submitted to the Genbank database. The accession numbers for the *groEL*- and *imp* sequences are MT638069 to MT638098 and MT668426 to MT668500, respectively. The *groEL*- and *imp* gene sequences of ‘*Ca.* P. ziziphi’ (CP025121) and ‘*Ca.* P. mali’ (CU469464) were extracted from the full genome sequences. Multiple alignments were performed using Clustal W^44^. The phylogenetic analysis of the nucleotide sequences and amino acids sequences was performed with MEGA X software^45^, using the maximum likelihood method and the complete deletion of positions containing gaps and missing data, and with a bootstrap value for 1000 replicates^46^. The phylogenetic analysis comprised 292 sequences for the *groEL* and 294 sequences for the *imp* genes. The database entries KJ939991 and KJ939992 of a partial *groEL* gene from 16SrV-group phytoplasmas, classified as ‘*Ca.* P. ulmi’, were included in a preliminary phylogenetic analysis. For *imp* tree construction, the database entries MK614707 (FD-D) and KJ402359 (FD-C Piemonte) were included. For clarity, the depicted phylogenetic trees were inferred with only one representative of each genotype group. The multiple sequence alignments of the *groEL*- and *imp* fragments were also used to calculate the ratio between non-synonymous and synonymous sites (dN/dS) which were calculated as an average over all codon sites in each gene using the Nei-Gojobori method^47^ with Jukes-Cantor model included in the MEGA X software package^45^.

### Distribution pattern of *groEL* and *imp* genotypes and gene linkage

Coordinates of individual trees were recorded in WGS84 format and imported to the software program BaseCamp version 4.7.1 (Garmin Ltd, Olathe). For a graphical display of spatial distribution, *groEL*- and *imp* genotypes were depicted as differently coloured dots on maps. *GroEL*- and *imp* sequences obtained from the same DNA extract were considered as linked when sequencing electropherograms gave no indication of polymorphic PCR products. Furthermore, it was assumed that primer sets amplified the genes of the prevalent ‘*Ca.* P. ulmi’ strain and that both genes were present in single copy.

## Supplementary Information

Supplementary Figure S1 Legend.

Supplementary Figure S2 Legend.

Supplementary Figure S1.

Supplementary Figure S2.

## Data Availability

Accession codes: DNA sequences determined in the course of this study have been deposited in GenBank (ncbi.nlm.nih.gov) under accession numbers MT638069 to MT638098 for the *groEL* fragments and MT668426 to MT668500 for the *imp* sequences.
