# Supplementary material for: Genetic variation, phylogenetic relationship and spatial distribution of ‘Candidatus Phytoplasma ulmi’ strains in Germany
Source: Sci Rep. 2020 Dec 14;10:21864. doi: 10.1038/s41598-020-78745-w (PMC7736341; doi:10.1038/s41598-020-78745-w)
Supplement: Supplementary file 1 — Supplementary Figure S1 Legend. [file 41598_2020_78745_MOESM1_ESM.docx]

Supplemetary Figure 1.

Genetic Variation, Phylogenetic Relationship and Spatial Distribution of *‘Candidatus* Phytoplasma ulmi’ Strains in Germany

B. Schneider, B. Hüttel, C. Zübert, and M. Kube

Supplementary Figure 1. Amplification of *imp* fragments from ‘*Ca.* P. ulmi’ accessions. Sample names are as following (left to right) including the abbreviation of the host plant and the Federal State; 0144_Ul_BY, 0162_Ug_BY, 0163_Ug_BY, 0491_Ul_BW, 0371_Ug_BB, 0622_Um_BW, 1031_Ul_BB, 2358_Ul_BB, 3555_Ug_NI, 5019_Uh_TH, ULW, Ug_h (DNA from a healthy Scots elm accession). The fragment length is about 600 base pairs. The gel was photographed with a VWR Imager 2 System, Software Version 1.5.6.0 (https://de.vwr.com).
