# Supplementary figures and images for: Genetic variation, phylogenetic relationship and spatial distribution of ‘Candidatus Phytoplasma ulmi’ strains in Germany
Source: Sci Rep. 2020 Dec 14;10:21864. doi: 10.1038/s41598-020-78745-w (PMC7736341; doi:10.1038/s41598-020-78745-w)

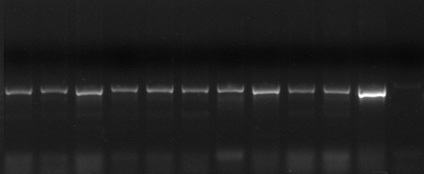

Supplement: Supplementary file 3 — Supplementary Figure S1. [file 41598_2020_78745_MOESM3_ESM.jpg]

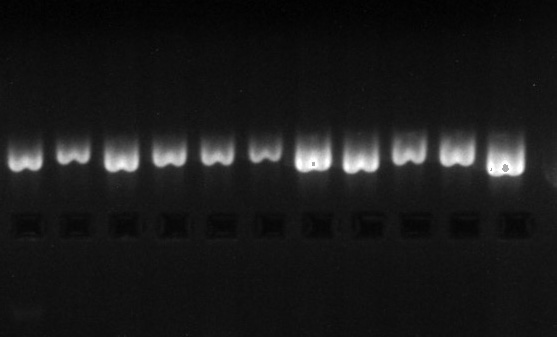

Supplement: Supplementary file 4 — Supplementary Figure S2. [file 41598_2020_78745_MOESM4_ESM.jpg]
